# Transcranial Direct Current Stimulation of the Temporoparietal Junction and Inferior Frontal Cortex Improves Imitation-Inhibition and Perspective-Taking with no Effect on the Autism-Spectrum Quotient Score

**DOI:** 10.3389/fnbeh.2017.00084

**Published:** 2017-05-09

**Authors:** Satoshi Nobusako, Yuki Nishi, Yuki Nishi, Takashi Shuto, Daiki Asano, Michihiro Osumi, Shu Morioka

**Affiliations:** ^1^Neurorehabilitation Research Center, Kio UniversityNara, Japan; ^2^Graduate School of Health Science, Kio UniversityNara, Japan; ^3^Department of Rehabilitation, Nishiyamato Rehabilitation HospitalOsaka, Japan; ^4^Department of Home-Visit Rehabilitation, Ishida ClinicOsaka, Japan; ^5^Department of Rehabilitation, Nogami HospitalOsaka, Japan; ^6^Department of Rehabilitation, Japan Baptist HospitalOsaka, Japan

**Keywords:** temporoparietal junction (TPJ), inferior frontal cortex (IFC), transcranial direct current stimulation (tDCS), imitation-inhibition, visual perspective-taking, autism-spectrum quotient (AQ)

## Abstract

Lesions to brain regions such as the temporoparietal junction (TPJ) and inferior frontal cortex (IFC) are thought to cause autism-spectrum disorder (ASD). Previous studies indicated that transcranial direct current stimulation (tDCS) of the right TPJ improves social cognitive functions such as imitation-inhibition and perspective-taking. Although previous work shows that tDCS of the right IFC improves imitation-inhibition, its effects on perspective-taking have yet to be determined. In addition, the role of the TPJ and IFC in determining the Autism-Spectrum Quotient (AQ), which is a measure of autism spectrum traits, is still unclear. Thus, the current study performed tDCS on the right TPJ and the right IFC of healthy adults, and examined its effects on imitation-inhibition, perspective-taking and AQ scores. Based on previous studies, we hypothesized that anodal tDCS of the right IFC and right TPJ would improve imitation-inhibition, perspective-taking and the AQ score. Anodal tDCS of the right TPJ or IFC significantly decreased the interference effect in an imitation-inhibition task and the cost of perspective-taking in a perspective-taking task, in comparison to the sham stimulation control. These findings indicated that both the TPJ and the IFC play a role in imitation-inhibition and perspective-taking, i.e., control of self and other representations. However, anodal stimulation of the right TPJ and the right IFC did not alter participants’ AQ. This finding conflicts with results from previous brain imaging studies, which could be attributed to methodological differences such as variation in sex, age and ASD. Therefore, further research is necessary to determine the relationship between the TPJ and IFC, and the AQ.

## Introduction

Previous research on autism-spectrum disorder (ASD) has suggested that it could be attributed to a “broken” mirror neuron system (MNS), of which the right inferior frontal cortex (IFC) is a core component. One piece of evidence substantiating this theory is that children with ASD have significantly less activity in the IFC while imitating and observing emotional expressions, in comparison to children with typical development (Dapretto et al., 2006). The same study noted a significant inverse correlation between the activity in the IFC and scores on the Autism Diagnostic Observation Schedule-Generic (ADOS-G) and Autism Diagnostic Observation Interview-Revised scales. The study in question used functional magnetic resonance imaging (fMRI) to gauge the level of MNS dysfunction in individuals with ASD. Others however, have used electroencephalography (Oberman et al., 2005; Bernier et al., 2007; Martineau et al., 2008), magnetoencephalography (Nishitani et al., 2004; Honaga et al., 2010), transcranial magnetic stimulation (Théoret et al., 2005; Enticott et al., 2012), and electromyography (Cattaneo et al., 2007).

Over the past few years, studies have cited that the dysfunctional control of the MNS that involves the medial prefrontal cortex (mPFC) and temporoparietal junction (TPJ) could be a potential pathogenic mechanism underlying ASD (Brass et al., 2009; Spengler et al., 2010; Wang et al., 2011; Spunt and Lieberman, 2012). An imitation-inhibition task allows a researcher to measure the functional control of the MNS through behavioral testing. There is also an increase in the interference effect of errors and reaction time (RT) in an imitation-inhibition task for individuals with ASD, in comparison to the control group (Spengler et al., 2010). The same study also noted a significant correlation between the interference effect of errors in the imitation-inhibition task and the RT on a theory of mind (ToM) task, as well as between the interference effect of errors and the scores on the ADOS scale. Furthermore, the interference effect of errors is inversely correlated with the activity in the mPFC and TPJ during a ToM task (Spengler et al., 2010). Another study also indicated that the interference effect of RT for individuals with ASD was greater than the control group, with a significant correlation between the interference effect of RT and the severity of ASD (ADOS score; Sowden et al., 2016).

Thus, there is evidence for the broken mirror theory and dysfunctional control of the MNS in ASD. However, the specific neuronal mechanisms responsible for ASD are still unclear. Over the past few years, studies have used neuromodulation techniques such as transcranial direct current stimulation (tDCS) to examine brain regions, which are associated with social cognitive function, that are damaged in individuals with ASD.

Santiesteban et al. (2012a) indicated that anodal tDCS of the right TPJ significantly decreased the interference effect of RT in an imitation-inhibition task and significantly increased accuracy during a perspective-taking task. Nevertheless, their performance on a self-referential task, which included components of a ToM task, were not significantly different between individuals who received anodal tDCS of the right TPJ, cathodal stimulation, or sham stimulation. Sowden and Catmur (2015) noted an increased effect on imitative compatibility, but not spatial compatibility, following stimulation of the right TPJ. Hogeveen et al. (2015) performed anodal tDCS of the right TPJ and IFC and noted a decrease in the interference effect during an imitation-inhibition task. However, there was an increase in face touching (imitation) during a social interaction task in the individuals who received anodal tDCS to the right IFC. Santiesteban et al. (2015) performed anodal tDCS on the right or left TPJ and reported a significant decrease in the interference effect of the inhibition of imitation, regardless of whether the right or left TPJ was stimulated. They also reported that accuracy during the visual perspective-taking task increased significantly, regardless of the side of the TPJ that was stimulated. However, they found that anodal tDCS of the right or left TPJ had no effect on performance of a ToM task. These studies have indicated that the right TPJ is involved in both imitation-inhibition, which requires enhancement of self-representation and inhibition of representations of the other, and perspective-taking, which requires inhibition of self-representation and enhancement of representations of the other. The right IFC is involved in both imitation and imitation-inhibition. However, these studies have shown that stimulation of either the right TPJ or the right IFC does not affect performance on a ToM task.

The right IFC is involved in both imitation and imitation-inhibition (Hogeveen et al., 2015). Imitation requires inhibition of self-representation and enhancement of the representation of the other. Therefore, anodal tDCS of the right IFC will presumably improve perspective-taking. This hypothesis is further strengthened by the fact that IFC plays a role in paying attention to others in social contexts (Kuang, 2016a), which is required in a perspective-taking task. Since social cognitive dysfunction is evident in autism spectrum traits, measured by Autism-Spectrum Quotient (AQ), if lesions to the IFC and TPJ are responsible for ASD, then modulation of the functioning of IFC and TPJ by tDCS could alter an individual’s AQ. In fact, previous studies suggested that the activity of TPJ and IFC is related to the total AQ score (Kosaka et al., 2010; Jung et al., 2014, 2015). Thus, the current study hypothesized that anodal tDCS of the right IFC would improve both imitation-inhibition and perspective-taking, while anodal tDCS of the right IFC and right TPJ would improve (i.e., lower) an individual’s AQ score.

To verify these hypotheses, anodal tDCS or sham stimulation was performed on the right TPJ and the right IFC of healthy adults, and its effect on imitation-inhibition, perspective-taking, and AQ scores were evaluated.

## Materials and Methods

### Participants

The study cohort comprised 30 young adults, 15 men and 15 women, aged 21.37 ± 1.22 years (mean age ± standard deviation [SD]), who were recruited from Kio University where they were enrolled as students. All participants were right-handed according to the Edinburgh Handedness Inventory (Oldfield, 1971), and none had a previous diagnosis of a developmental disorder, or physical or mental disability. All participants were familiar with operating a personal computer.

The 30 participants were divided into three groups: the sham group (*n* = 10), or experimental groups, where tDCS was applied to the TPJ (*n* = 10) or IFC (*n* = 10). Each group was matched in terms of gender (with five men and five women in each group; *χ*^2^ = 0.000, $\chi_{\text{(0.95)}}^{\text{2}}$ = 5.991, *p* = 1.000) and age (TPJ: 21.20 ± 0.75 years; IFC: 21.50 ± 1.36 years; sham: 21.40 ± 1.43 years; *p* = 0.970).

The Ethics committee of the Graduate School and Faculty of Health Sciences at Kio University approved (approval number: H27-33) the experimental procedures. Participants provided background information and gave written informed consent. All of the participants read a tDCS information sheet and verified that they did not display any contraindications to tDCS. The procedures complied with the ethical standards of the 1964 Declaration of Helsinki regarding the treatment of human participants in research. There were no foreseeable risks to the participants, and no personally identifying information was collected.

### Procedures

After receiving tDCS, each participant performed two behavioral tasks (an imitation-inhibition task and visual perspective-taking task) and completed the AQ questionnaire. Each of the three tasks were performed in random order.

### Transcranial Direct Current Stimulation Procedures

The tDCS was performed as previously described (Hogeveen et al., 2015). Briefly, tDCS was delivered through a pair of 35 cm^2^ sponge electrodes, which were soaked in saline and connected to a neuroConn DC-stimulator Plus (neuroConn, Ilmenau, Germany). Stimulation sites for the tDCS protocol were identified using an EasyCap landmark cap (EasyCap, Herrsching, Germany), which were modified according to standard 10% landmarks. Previous studies primarily implicated a role for right-lateralized TPJ activity (Brass et al., 2005, 2009) and bilateral IFC activity (Brass et al., 2005; Cross et al., 2013) in the control of imitation. Further, Hogeveen et al. (2015) performed tDCS of the TPJ and IFC of the right hemisphere. Thus, anodal tDCS was applied to the right TPJ or IFC in the current study. The stimulation sites for the IFC and TPJ were FC6 (Holland et al., 2011; Hogeveen et al., 2015) and CP6 (Santiesteban et al., 2012a, 2015; Hogeveen et al., 2015), respectively. The reference electrode was placed horizontally over the vertex, individually measured, and then the vertex at 50% of the distance between the preauricular points, crossing a point 50% of the distance between the inion and nasion, was marked. For the TPJ stimulation, the anodal electrode was placed at CP6 with the cathodal electrode at the vertex. For the IFC stimulation, the anodal electrode was placed at FC6 with the cathodal electrode at the vertex. Both patterns of electrode placement were equally used for sham stimulation. The placement of these electrodes was the same as in previous studies (Santiesteban et al., 2012a, 2015; Hogeveen et al., 2015). For active tDCS, stimulation began with a 15-s ramp-up to 1 mA, proceeded to stimulation at 1 mA for 20 min, and ended with a 15-s ramp-down period. For sham stimulation, the same ramping procedure was accompanied by a 30 s stimulation period, yet participants were left in the room for the same total duration to mimic the experience of real stimulation without any neuromodulatory effect (Gandiga et al., 2006; Nitsche et al., 2008). During the stimulation period, participants were instructed to sit quietly with their eyes close and to think of nothing in particular, in order to minimize any attention to environmental stimuli (Damoiseaux et al., 2006; Tambini et al., 2010). Following tDCS stimulation, patients completed the imitation-inhibition task within 15 min, the perspective-taking task within 15 min, and the AQ within 10 min. The total time from the start of tDCS to the completion of all three tasks were less than 1 h. Previous studies using measures of corticospinal excitability have suggested that the neuromodulatory effects of 13 min of active tDCS are robust for 90 min post-stimulation (Nitsche and Paulus, 2001), suggesting that the current procedures were completed within the critical window.

### Experimental Tasks and Questionnaire

#### Imitation-Inhibition Task

**Figure 1:**
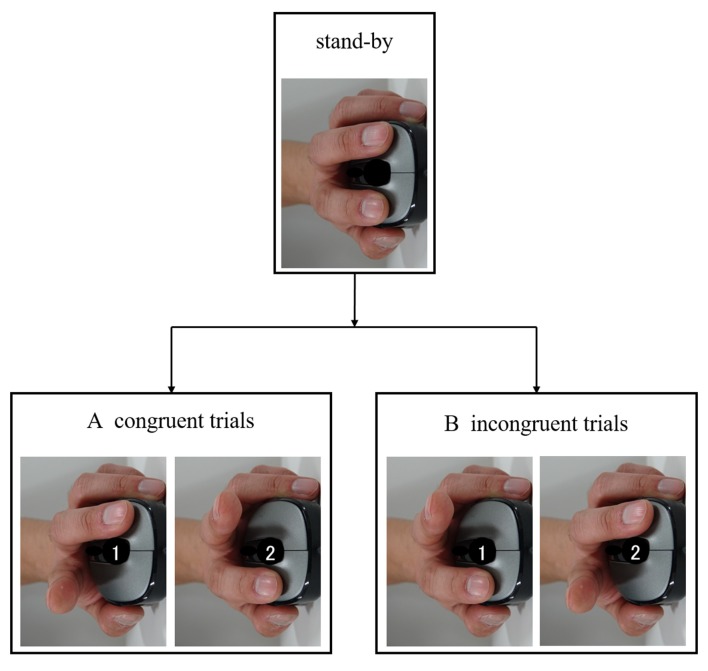
**Imitation-inhibition task.** Participants were shown a video of a demonstrator’s left hand. In accordance with the number (cue) shown, participants lifted either their index (1) or middle finger (2) of their right hand. **(A)** In congruent trials, participants were instructed to lift the same finger as lifted by the hand shown in the video. **(B)** In incongruent trials, participants were instructed to lift a finger other than that lifted by the hand shown in the video.

Participants performed an imitation-inhibition task based on the ones designed by Brass et al. (2000), Santiesteban et al. (2012a), Hogeveen et al. (2015), and Santiesteban et al. (2015). The stimulus consisted of a brief video showing the demonstrator’s left hand from a third person point of view (Figure 1). The hand lifted either its index or middle finger. Stimuli were presented on a 13.3-inch monitor (NEC; screen resolution 2560 × 1440 pixels). Participants were first shown a standby frame (no number cue, 2000 ms) with the hand resting on a mouse, followed by the video and a number. Following participant reaction, a black screen was then displayed until the next standby frame was shown (Figure 1). The demonstrator’s left hand was rotated around the sagittal and transverse planes with respect to the participant’s right hand, which rested on a mouse. As response movements were spatially orthogonal to stimulus movements, imitation was isolated from spatial compatibility. In accordance with the number (cue) shown, participants lifted either their index (1) or middle finger (2) of their right hand. The task consisted of two trials. In congruent trials, participants were instructed to lift the same finger as the video (Figure 1A), while in incongruent trials, participants were instructed to lift a different finger to that shown in the video (Figure 1B). Thus, incongruent trials required participants to inhibit their automatic imitation response to perform the instructed action. After 20 practice trials, 40 trials were conducted with random depictions of four combinations of conditions (two displayed numbers and lifting of the index or middle finger). The time from when the video was shown until the participant reacted by lifting a finger from the mouse served as the RT, which was recorded. The accuracy and error rate (ER) of the responses were also recorded. Accuracy and ER were recorded based on the response when a participant lifted his or her index or middle finger. The task was created, implemented and recorded using Super Lab 5 (Cedrus Corporation, San Pedro, CA, USA).

#### Visual Perspective-Taking Task

**Figure 2:**
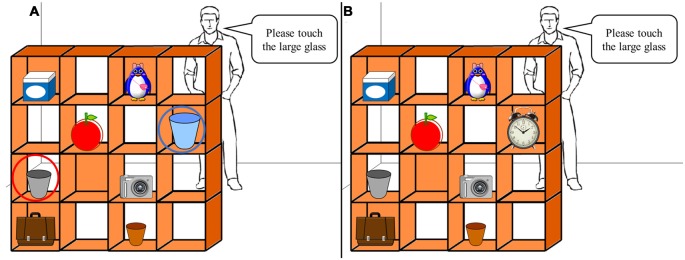
**Perspective-taking task. (A)** Example of an experimental trial requiring participants to inhibit the “self” perspective (blue circle) and adopt the perspective of the “other” (red circle). When instructed to touch the large glass, participants had to ignore the largest glass they saw and choose the medium-sized glass that the “other” can see. The red and blue circles were not displayed during the task. **(B)** Example of the control trials where the self and other perspectives were not in conflict (same instruction as **A**).

A computerized version of the original task described by Santiesteban et al. (2012a, 2015) was used. The task, which was initially developed by Keysar et al. (2000), required participants to take the viewpoint of a character, i.e., “the director”. The visual stimuli consisted of a 4 × 4 grid (“shelves”) containing eight different objects. Five slots were occluded from the director’s view (Figure 2) and participants were instructed to touch the object specified by the director. During the experimental trials, there was a conflict between the participant’s and the director’s perspective. If, for example, the participant was presented with the array shown in Figure 2A, they had to ignore “competitor object”, which the director could not see, and pick the next eligible object that was visible to the director. Under control conditions, an irrelevant object replaced the competitor item from the experimental conditions, but instructions remained the same (Figure 2B). There were 10 experimental conditions and 10 control conditions, with 20 trials in total. The conditions were shown in a random order. Accuracy of the selection and movement of the target object and RT were recorded. The task was created, implemented and recorded using LabVIEW (National Instruments, Austin, TX, USA).

#### Autism-Spectrum Quotient

The AQ is a brief, self-administered questionnaire that was developed by Baron-Cohen et al. (2001) to measure personality traits associated with the autistic spectrum in adults of typical intelligence (Supplementary Material). Wakabayashi et al. (2004) created a Japanese version of the AQ that was used in the current study. The AQ consists of 50 statements rated on a 4-point Likert scale (where 1 = definitely agree and 4 = definitely disagree). Half of the statements are worded to elicit a “disagree” response and half are worded to elicit an “agree” response. The AQ allows discernment of multiple aspects that characterize autism, such as social skills, attention switching, attention to detail, communication and imagination (Rutter, 1978; Wing and Gould, 1979). The total AQ score ranges from a minimum of 0 points to a maximum of 50 points. The AQ has good internal consistency and construct validity, strong test-retest and inter-rater reliability and robust self-vs.-parent-report reliability (Baron-Cohen et al., 2001). In addition, the AQ is inversely correlated with both the Friendship and Relationship Quotient (Baron-Cohen and Wheelwright, 2003) and the Empathy Quotient (Baron-Cohen and Wheelwright, 2004), and is correlated with the Systemizing Quotient and Systemizing Quotient-Revised (Baron-Cohen et al., 2003; Wheelwright et al., 2006). The AQ is strongly predictive of individuals who will be diagnosed with ASD in a clinical setting (Woodbury-Smith et al., 2005). The AQ has also been found to reflect sex differences (males > females) and cognitive differences (scientists > nonscientists; Baron-Cohen et al., 2001). This pattern of results has been closely replicated in a Japanese sample (Wakabayashi et al., 2004). Thus, the AQ was deemed the most appropriate way to measure autistic traits modulated by tDCS in the current study.

### Data Analysis

#### Imitation-Inhibition Task

Like previous studies (Santiesteban et al., 2012a, 2015; Hogeveen et al., 2015), the current study used task data to determine the accuracy rate and RT during congruent trials and incongruent trials. In those previous studies, the ER and RT during incongruent trials served as an indicator of the ability to inhibit imitation. The ER and RT during congruent trials were subtracted from the ER and RT during incongruent trials to calculate the interference effect of the inhibition of imitation (incongruent-congruent).

The Shapiro-Wilk test revealed no normality in the accuracy rate during congruent trials and incongruent trials and the interference effect of ER. Thus, a Kruskal-Wallis test was used to compare the three groups. The Mann-Whitney U test, with Bonferroni correction, was used for *post hoc* analysis. The current study compared results among the three groups and the significance level was set at *P* < 0.016. The accuracy rate during congruent trials and incongruent trials were compared (within groups) using the Wilcoxon signed-rank test. The significance level was set at *P* < 0.05.

The Shapiro-Wilk test revealed normality in the RT during congruent trials and incongruent trials and the interference effect of RT. Thus, analysis of variance (ANOVA) was performed on between-group factors (the TPJ group, the IFC group and the sham group) and between-trial factors (congruent trials and incongruent trials) based on a split-plot factorial design. In addition, one-way ANOVA was used to compare the interference effect of RT among the groups, and Tukey’s test was used as a *post hoc* test. The significance level was set at *P* < 0.05.

#### Visual Perspective-Taking Task

Like previous studies (Santiesteban et al., 2012a,b, 2015), the current study used task data to determine the accuracy rate and RT under experimental conditions (perspective-taking) and control conditions. Previous studies (Keysar et al., 2000; Santiesteban et al., 2012a,b, 2015) noted an increase in the ER and a delay in the RT under experimental conditions in comparison to the ER and RT under control conditions. In the current study, the ER and RT under control conditions were subtracted from the ER and RT under experimental conditions to calculate the cost of perspective-taking.

The Shapiro-Wilk test revealed no normality in the accuracy rate and RT under experimental conditions and control conditions and the cost of perspective-taking (ER, RT). Thus, a Kruskal-Wallis test was used to compare the three groups. The Mann-Whitney U test was used for *post hoc* analysis and Bonferroni correction was used to adjust the *p*-values obtained in *post hoc* analyses. The current study compared results among the three groups and the significance level was set at *P* < 0.016.

The accuracy rate and RT under experimental conditions and control conditions were compared (within groups) using the Wilcoxon signed-rank test. The significance level was set at *P* < 0.05.

#### AQ

The Shapiro-Wilk test revealed no normality in the total AQ score and the scores on the five subscales of the AQ, so groups were compared using the Kruskal-Wallis test. The Mann-Whitney U test with Bonferroni correction was used to compare results between the three groups. The significance level was set at *P* < 0.016. All statistical analyses were performed using SPSS ver. 24 (SPSS, Chicago, IL, USA).

## Results

### Imitation-Inhibition Task

**Figure 3:**
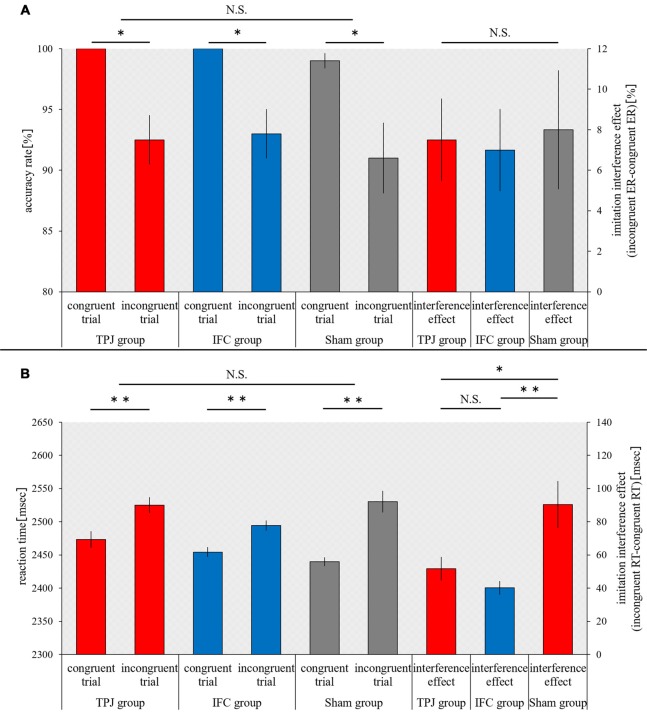
**Results of the imitation-inhibition task.** The horizontal axis shows each trial and interference effects in each group. **(A)** The mean accuracy rate and interference effect (error rate (ER)) during each trial for each group. **(B)** The mean reaction time (RT) and interference effect (RT) during each trial for each group. Red bars, temporoparietal junction (TPJ) group; Blue bars, inferior frontal cortex (IFC) group; Gray bars, Sham group. Error bars represent the standard error of the mean (SEM). **p < 0.01; *p < 0.05; N.S. not significant.

The accuracy rate during congruent trials and incongruent trials, and the interference effect of ER were not significantly different between the groups (congruent trials, *p* = 0.126; incongruent trials, *p* = 0.961; ER, *p* = 0.961, Kruskal-Wallis test). In comparison to the accuracy rate for congruent trial, the accuracy of incongruent trials significantly decreased for all three groups (TPJ group, *p* = 0.017; IFC group, *p* = 0.017; sham group, *p* = 0.016; Figure 3A).

Based on a split-plot factorial design, an ANOVA analysis of the effect of between-group factors and between-trial factors on the RT revealed a main effect of between-trial factors (*F*_(1,27)_ = 111.936, *p* = 0.000, $\eta_{\text{p}}^{\text{2}}$ = 0.806) and interaction effect (*F*_(2,27)_ = 7.019, *p* = 0.004, $\eta_{\text{p}}^{\text{2}}$ = 0.342). A main effect of between-group factors was not noted (*F*_(2,27)_ = 1.416, *p* = 0.260, $\eta_{\text{p}}^{\text{2}}$ = 0.095). A simple main effect analysis (with Bonferroni adjustment) revealed a significant increase in the RT for all three groups (*p* = 0.000 for all) during incongruent trials, in comparison to the RT during congruent trials. In addition, a simple main effect analysis (with Bonferroni adjustment) revealed no differences in the RT among groups during individual trials for congruent trials (TPJ vs. IFC, *p* = 0.548; TPJ vs. sham, *p* = 0.067; and IFC vs. sham, *p* = 0.902) and incongruent trials (TPJ vs. IFC, *p* = 0.332; TPJ vs. sham, *p* = 1.000; and IFC vs. sham, *p* = 0.190). A one-way ANOVA revealed significant differences in the interference effect of RT among groups (*F*_(2,29)_ = 7.019, *p* = 0.004). A multiple comparison test using Tukey’s method revealed that the interference effect of RT decreased significantly for the TPJ group and the IFC group, in comparison to the sham group (TPJ vs. sham, *p* = 0.027 and IFC vs. sham, *p* = 0.004). Significant differences in the interference effect of RT were not noted for the TPJ group and the IFC group (*p* = 0.693; Figure 3B).

### Visual Perspective-Taking Task

**Figure 4:**
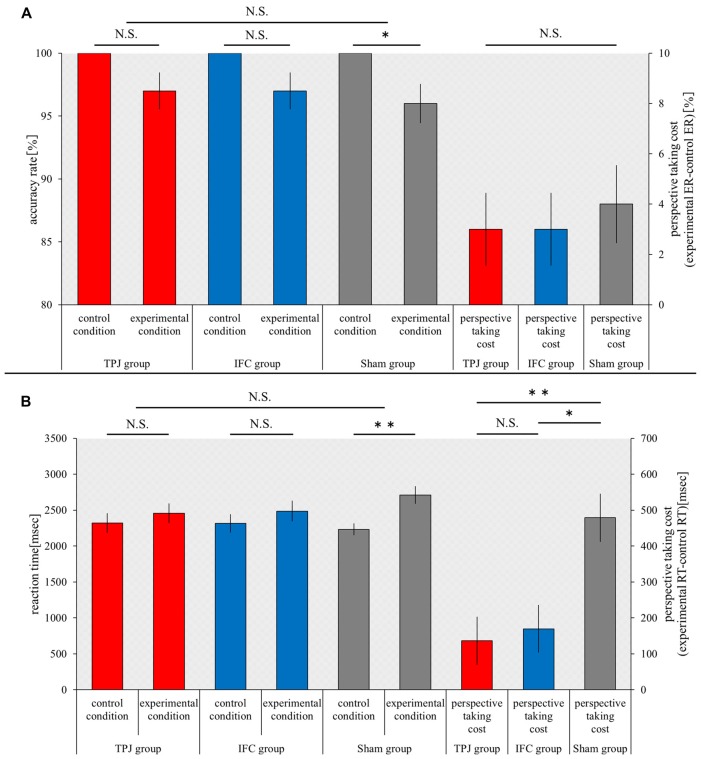
**Results of the visual perspective-taking task.** The horizontal axis shows each condition and the cost of perspective-taking for each group. **(A)** The mean accuracy rate under each condition for each group and the cost of perspective-taking (ER) for each group. **(B)** The mean RT under each condition for each group and the mean the cost of perspective-taking (RT) for each group. Red bars, TPJ group; Blue bars, IFC group; Gray bars, Sham group. Error bars represent the SEM. **p < 0.01; *p < 0.05; N.S. not significant.

The accuracy rate under control conditions and experimental conditions and the cost of perspective-taking (ER) were compared between groups using a Kruskal-Wallis test. There was no significant difference in the accuracy rate under control conditions and experimental conditions and the cost of perspective-taking (control conditions, *p* = 1.000; experimental conditions, *p* = 0.865; and the cost of perspective-taking, *p* = 0.865). The accuracy rate under control conditions and experimental conditions, which was compared (within groups) using a Wilcoxon signed-rank test, was not significantly different in the TPJ group or IFC group (*p* = 0.083 for both). However, a significant decrease in the accuracy rate under experimental conditions was noted in the sham group, when compared to the accuracy rate under control conditions (*p* = 0.046; Figure 4A).

The RT under control conditions and experimental conditions and the cost of perspective-taking (RT), which were compared between groups using the Kruskal-Wallis test, were not significantly different (control conditions, *p* = 0.966; experimental conditions, *p* = 0.282). However, significant differences in the cost of perspective-taking were noted among the three groups (*p* = 0.001). The Mann-Whitney U test with Bonferroni p-correction (*p* < 0.016) was performed as a *post hoc* test. Results revealed a significant decrease in the cost of perspective-taking in the TPJ group in comparison to the sham group (*p* = 0.000). A significant decrease in the cost of perspective-taking was also noted in the IFC group in comparison to the sham group (*p* = 0.005). Significant differences in the cost of perspective-taking were not noted for the TPJ group or IFC group (*p* = 0.579). The RT under control conditions and experimental conditions was compared (within groups) using the Wilcoxon signed-rank test, and revealed no significant differences in the TPJ and IFC groups (TPJ group, *p* = 0.114; IFC group, *p* = 0.093). However, a significant increase in the RT was noted in the sham group under experimental conditions, in comparison to control conditions (*p* = 0.005; Figure 4B).

### Autism-Spectrum Quotient

**Figure 5:**
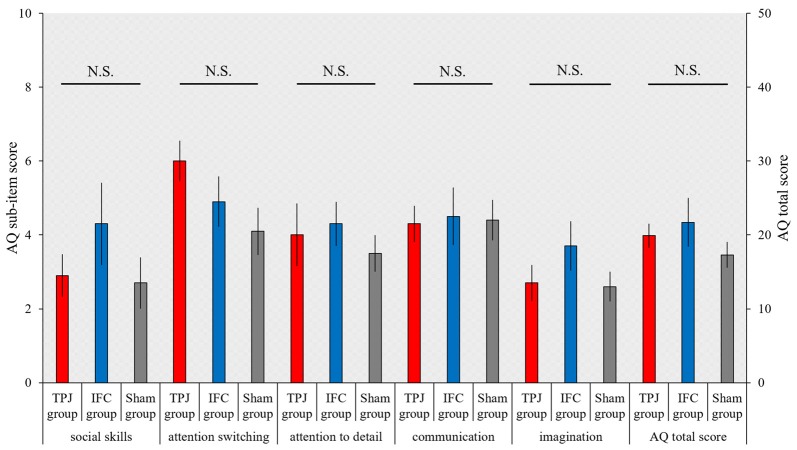
**Results of the Autism-Spectrum Quotient (AQ).** The mean AQ scores for each group. The horizontal axis shows scores on AQ subscales and the total AQ score for each group. Red bars, TPJ group; Blue bars, IFC group; Gray bars: Sham group. Error bars represent the SEM.

The total AQ score and scores on the five AQ subscales, which were compared between groups using the Kruskal-Wallis test, was not significantly different (total AQ score, *p* = 0.527; social skills, *p* = 0.711; attention switching, *p* = 0.154; attention to detail, *p* = 0.633; communication, *p* = 0.963; imagination, *p* = 0.441; Figure 5).

## Discussion

### Imitation-Inhibition Task

The interference effect of RT decreased significantly in the TPJ group in comparison to the sham group, in line with several previous studies (Santiesteban et al., 2012a, 2015; Hogeveen et al., 2015; Sowden and Catmur, 2015). The current results support a role for the right TPJ in controlling self and other representations, i.e., it inhibits imitation of others and it enhances one’s ability to control oneself.

Further, the interference effect of RT decreased significantly in the IFC group in comparison to the sham group, which corroborates with a previous study (Hogeveen et al., 2015). Hogeveen et al. (2015) indicated that anodal tDCS of the right TPJ improved the inhibition of imitation, but it did not facilitate imitation. Further, anodal tDCS of the right IFC improved both inhibition of imitation and imitation. These finding indicate that the right TPJ is indirectly involved in controlling imitation, while the right IFC is directly involved in controlling imitation (Hogeveen et al., 2015). The results from the current study support a role for the right IFC in directly inhibiting imitation.

### Visual Perspective-Taking Task

The cost of RT for perspective-taking decreased significantly in the TPJ group in comparison to the sham group. For the sham group, the accuracy rate significantly decreased under experimental conditions, while the RT significantly increased. However, there were no significant differences in the accuracy rate and the RT under experimental conditions in the TPJ group, which is similar to previous studies (Santiesteban et al., 2012a, 2015). There is ample evidence that the TPJ plays a role in social cognition, i.e., social moral judgment, hostile intention attribution, out-group punishment, false-belief and mentalizing (Donaldson et al., 2015). The results from the current study support the role of the right TPJ in inhibiting self-representation and enhancing representations of the other during perspective-taking.

A principal aim of the current study was to examine the effects of anodal tDCS of the right IFC on perspective-taking. The cost of RT for perspective-taking significantly decreased in the IFC group, in comparison to the sham group. For the sham group, the accuracy rate under experimental conditions significantly decreased, compared to that under control conditions. Further, the RT under experimental conditions significantly increased, compared to that under control conditions. However, there were no significant differences in the accuracy rate and the RT under experimental and control conditions in the IFC group.

Previous studies have noted increased brain activity in the right IFC during the visual perspective-taking task (Kaiser et al., 2008) and mental rotation of an object (Yassa et al., 2008; Hattemer et al., 2011; Semrud-Clikeman et al., 2012). Thus, the IFC group should have improved their ability to rotate an object mentally, so the group may be better able to perform a mental activity like taking the perspective of the director in the perspective-taking task.

Using fMRI, Mazzarella et al. (2013) demonstrated that the dorsomedialPFC was sensitive to the orientation of the actor in the altercentric task (i.e., object identification from an actor’s perspective), while the right IFC was sensitive to the orientation of the actor in the egocentric task (i.e., target object identification from their own perspective). Further, the dorsomedialPFC and the right IFC may play distinct but complementary roles in visual perspective-taking (Mazzarella et al., 2013). These findings suggested that anodal tDCS of the right IFC might facilitate awareness of the orientation of the director in the current study as well.

Hogeveen et al. (2015) indicated that anodal tDCS of the right IFC improves both imitation and inhibition of imitation. This finding indicates that the right IFC plays a role in inhibiting self-representation and enhancing representations of the other, and vice versa. Thus, the right IFC may facilitate inhibition of imitation, which requires enhancement of self-representation and inhibition of representations of the other, as well as perspective-taking, which requires inhibition of self-representations and enhancement of representations of the other. Although there may be other factors involved, the current study highlighted the role the right IFC plays in perspective-taking.

Further, both the TPJ and IFC are brain regions that play a role in being attentive to others in a social context (Kuang, 2016a). Perspective-taking requires an individual to direct attention away from oneself and towards others. Thus, stimulation of the TPJ and IFC could have improved attention to others.

### Both the Temporoparietal Junction and Inferior Frontal Cortex Contributed to Imitation-Inhibition and Perspective-Taking

The current study indicated that both the TPJ and IFC equally help to control different representations of self and others, i.e., inhibiting imitation of and taking the perspective of another. Kuang (2016b) states that instead of competing during action recognition during social situations, the mirror neuron (IFC) and mentalizing systems (TPJ) work in a synergistic and complementary manner to ensure appropriate social interactions in a given behavioral context. In other words, the dichotomy between mirroring and mentalizing processes suggests that the distinction between self and other operates at both the mental and physical level. Mirroring processes play a role in self-awareness and empathy, while mentalizing processes support bodily self-consciousness, i.e., a sense of body ownership and a sense of agency. The results from the current study support the theory that the self-other distinction is both mental (mirroring processes) and physical (mentalizing processes). The current results also indicate that either dysfunction of the IFC (the mirroring neuron system) or the TPJ (the mentalizing system) may lead to social-cognitive abnormalities, such as those seen in ASD.

### Autism-Spectrum Quotient

The AQ reflects autism spectrum traits. Since dysfunction of the TPJ or IFC can cause ASD, we hypothesized that anodal tDCS of these brain regions would reduce an individual’s AQ score. However, tDCS did not affect the total AQ scores or any of the scores on its five subscales.

Previous studies, using MRI/fMRI, suggested that the activity of TPJ and IFC is related to the AQ score. A study on pervasive developmental disorders in adults reported that the AQ score and gray matter volume of the IFC are inversely correlated (Kosaka et al., 2010). In typically developing male adults, the resting-state functional connectivity of the default mode network (which includes the TPJ) and the AQ score is inversely correlated (Jung et al., 2014, 2015). Thus, results from the current study conflict with these previous studies. However, this discrepancy could be attributed to the difference in the methodology (i.e., tDCS vs. fMRI).

The AQ score and performance on a ToM task are significantly correlated (Carroll and Chiew, 2006). However, a study using fMRI indicated that the TPJ is part of the core network for ToM (Schurz et al., 2014), while Santiesteban et al. (2012a), found that anodal tDCS of the right TPJ produced no changes in the RT on a self-referential task, which included components of a ToM task. Further, anodal tDCS of the right and left TPJ produced no changes in accuracy on a ToM task (Santiesteban et al., 2015). Santiesteban et al. (2015) posited that a ToM task is insensitive to performance variation induced by stimulation in typical development adults, but that clinical populations would exhibit marked deficits in ToM. The AQ score is similarly less susceptible to variability due to transcranial stimulation. Although the imitation-inhibition task and perspective-taking task measure on-line social-cognitive processing, the AQ score reflects metacognition and memory based on previous social interactions. Therefore, the AQ is a “self” assessment of one’s social cognitive or social behavioral traits. Thus, the results in the current study suggested that neuromodulation is only effective on the functioning of single brain regions, with no effect on metacognition or memory about one’s mental state. However, this is purely speculative, and the limitations outlined in the next section must also be taken into account. The current study was unable to ascertain the relationship between the TPJ and IFC, and the AQ score, and thus, further work is required to ascertain these associations.

### Limitations of the Current Study and Future Directions

The current study had a number of limitations. The placement of tDCS electrodes could not be adjusted enough. In addition, control tasks, such as those not involving inhibition of imitation, were not performed to assess the duration of the modulatory effect of tDCS, which may explain why it did not appear to affect the AQ score. Although each participant performed the three tasks after tDCS in a random order, the performance on the imitation-inhibition task and the perspective-taking task significantly improved in the TPJ and IFC stimulation groups. Thus, although these technical limitations presumably had little effect, these issues need to be resolved in future studies.

In addition, the current study did not use an intra-subject design. In other words, the three types of stimulation, i.e., TPJ, IFC and sham stimulations, were not counterbalanced across subjects, which might have had better explanatory power and less noise. Nevertheless, the current study was designed as a comparison of unpaired groups in order to eliminate the effects of learning due to repetition of tasks. In fact, previous studies have used a similar design for the same reason (Santiesteban et al., 2012a, 2015; Hogeveen et al., 2015). Eliminating the effects of learning isolated the simple modulatory effect of tDCS.

The current study involved an extremely small sample of 10 subjects per group (5 men and 5 women in each). Thus, the modulatory effect of tDCS may not have been sufficient to affect the AQ score. ASD is more common in men than women (Kirkovski et al., 2013; Ruigrok et al., 2014; Van Wijngaarden-Cremers et al., 2014), and men generally have higher AQ scores in the general population and the highest individual ASD scores of all participants (Baron-Cohen et al., 2001). Further, although men showed inverse correlation between the resting-state functional connectivity of the default mode network and AQ score, this correlation was not present in women (Jung et al., 2015). Thus, in future studies, the sample size needs to be substantially increased, so that the effects tDCS of the TPJ and IFC have on the AQ score can be separately examined in men and women.

Since the participants in the current study were typically developing adults without ASD, these results are not reproducible in individuals with ASD or in different age ranges. Thus, future studies need to involve participants who are typically developing, have ASD and belong to different age groups.

In addition, the current study merely verified the immediate effects of tDCS. Studies in the area of rehabilitation have indicated that continuous tDCS improved motor function (Costa-Ribeiro et al., 2016; Ilić et al., 2016; Yozbatiran et al., 2016; Figlewski et al., 2017). Thus, continuous tDCS is likely to affect an individual’s AQ score.

Finally, the AQ measure has five subscales. Currently, the relationship between individual scores of AQ and brain region/neural network is unknown. Thus, brain regions and neural networks that correlate with scores on the subscales of the AQ need to be examined using neuroimaging techniques such as fMRI and electroencephalography. Those findings would presumably lead to the development of treatment strategies for ASD as a whole, as well as strategies targeting individual symptoms of ASD.

## Conclusion

The current study indicated that anodal tDCS of the right TPJ or the right IFC immediately increased imitation-inhibition, which requires enhancement of self-representations and inhibition of representations of the other, as well as perspective-taking, which requires inhibition of self-representations and enhancement of representations of the other. However, the current results revealed that anodal tDCS of the right TPJ and the right IFC did not immediately affect an individual’s AQ. Further research is required to ascertain the relationship between the TPJ and IFC, and AQ.

## Authors Contributions

SN collected and analyzed the data, and wrote the manuscript. YN, YN and TS assisted in collecting data. SN, MO and DA designed the study. SM designed and supervised the study. All authors read and approved the manuscript.

## Funding

This work was supported by JSPS KAKENHI, Grant-in-Aid for Young Scientists (B) (Grant Number 16K16453).

## Conflict of Interest Statement

The authors declare that the research was conducted in the absence of any commercial or financial relationships that could be construed as a potential conflict of interest.
