# Supplementary material for: Transcranial Direct Current Stimulation of the Temporoparietal Junction and Inferior Frontal Cortex Improves Imitation-Inhibition and Perspective-Taking with no Effect on the Autism-Spectrum Quotient Score
Source: Front Behav Neurosci. 2017 May 9;11:84. doi: 10.3389/fnbeh.2017.00084 (PMC5422472; doi:10.3389/fnbeh.2017.00084)
Supplement: Supplementary file 1 [file Data_sheet_1.docx]

Supplementary Material

The Adult Autism Spectrum Quotient (AQ)

**For full details, please see:**

S. Baron-Cohen, S. Wheelwright, R. Skinner, J. Martin and E. Clubley, (2001)
[The Autism Spectrum Quotient (AQ) : Evidence from Asperger Syndrome/High Functioning Autism, Males and Females, Scientists and Mathematicians](javascript:PopUpViewDoc('The%20Autism%20Spectrum%20Quotient%20(AQ)%20:%20Evidence%20from%20Asperger%20Syndrome/High%20Functioning%20Autism,%20Males%20and%20Females,%20Scientists%20and%20Mathematicians','2001_BCetal_AQ.pdf'))
Journal of Autism and Developmental Disorders 31:5-17

| 1. | I prefer to do things with others rather than on my own. | definitely  agree | slightly  agree | slightly  disagree | definitely  disagree |
| --- | --- | --- | --- | --- | --- |
| 2. | I prefer to do things the same way over and over again. | definitely  agree | slightly  agree | slightly  disagree | definitely  disagree |
| 3. | If I try to imagine something, I find it very easy to create a picture in my mind. | definitely  agree | slightly  agree | slightly  disagree | definitely  disagree |
| 4. | I frequently get so strongly absorbed in one thing that I lose sight of other things. | definitely  agree | slightly  agree | slightly  disagree | definitely  disagree |
| 5. | I often notice small sounds when others do not. | definitely  agree | slightly  agree | slightly  disagree | definitely  disagree |
| 6. | I usually notice car number plates or similar strings of information. | definitely  agree | slightly  agree | slightly  disagree | definitely  disagree |
| 7. | Other people frequently tell me that what I have said is impolite, even though I think it is polite. | definitely  agree | slightly  agree | slightly  disagree | definitely  disagree |
| 8. | When I am reading a story, I can easily imagine what the characters might look like. | definitely  agree | slightly  agree | slightly  disagree | definitely  disagree |
| 9. | I am fascinated by dates. | definitely  agree | slightly  agree | slightly  disagree | definitely  disagree |
| 10. | In a social group, I can easily keep track of several different people’s conversations. | definitely  agree | slightly  agree | slightly  disagree | definitely  disagree |
| 11. | I find social situations easy. | definitely  agree | slightly  agree | slightly  disagree | definitely  disagree |
| 12. | I tend to notice details that others do not. | definitely  agree | slightly  agree | slightly  disagree | definitely  disagree |
| 13. | I would rather go to a library than a party. | definitely  agree | slightly  agree | slightly  disagree | definitely  disagree |
| 14. | I find making up stories easy. | definitely  agree | slightly  agree | slightly  disagree | definitely  disagree |
| 15. | I find myself drawn more strongly to people than to things. | definitely  agree | slightly  agree | slightly  disagree | definitely  disagree |
| 16. | I tend to have very strong interests, which I get upset about if I cannot pursue. | definitely  agree | slightly  agree | slightly  disagree | definitely  disagree |
| 17. | I enjoy social chitchat. | definitely  agree | slightly  agree | slightly  disagree | definitely  disagree |
| 18. | When I talk, it is not always easy for others to get a word in edgeways. | definitely  agree | slightly  agree | slightly  disagree | definitely  disagree |
| 19. | I am fascinated by numbers. | definitely  agree | slightly  agree | slightly  disagree | definitely  disagree |
| 20. | When I am reading a story, I find it difficult to work out the characters’ intentions. | definitely  agree | slightly  agree | slightly  disagree | definitely  disagree |
| 21. | I do not particularly enjoy reading fiction. | definitely  agree | slightly  agree | slightly  disagree | definitely  disagree |
| 22. | I find it hard to make new friends. | definitely  agree | slightly  agree | slightly  disagree | definitely  disagree |
| 23. | I notice patterns in things all the time. | definitely  agree | slightly  agree | slightly  disagree | definitely  disagree |
| 24. | I would rather go to the theatre than a museum. | definitely  agree | slightly  agree | slightly  disagree | definitely  disagree |
| 25. | It does not upset me if my daily routine is disturbed. | definitely  agree | slightly  agree | slightly  disagree | definitely  disagree |
| 26. | I frequently find that I do not know how to keep a conversation going. | definitely  agree | slightly  agree | slightly  disagree | definitely  disagree |
| 27. | I find it easy to “read between the lines” when someone is talking to me. | definitely  agree | slightly  agree | slightly  disagree | definitely  disagree |
| 28. | I usually concentrate more on the whole picture, rather than the small details. | definitely  agree | slightly  agree | slightly  disagree | definitely  disagree |
| 29. | I am not very good at remembering phone numbers. | definitely  agree | slightly  agree | slightly  disagree | definitely  disagree |
| 30. | I do not usually notice small changes in a situation, or a person’s appearance. | definitely  agree | slightly  agree | slightly  disagree | definitely  disagree |
| 31. | I know how to tell if someone listening to me is getting bored. | definitely  agree | slightly  agree | slightly  disagree | definitely  disagree |
| 32. | I find it easy to do more than one thing at once. | definitely  agree | slightly  agree | slightly  disagree | definitely  disagree |
| 33. | When I talk on the phone, I am not sure when it is my turn to speak. | definitely  agree | slightly  agree | slightly  disagree | definitely  disagree |
| 34. | I enjoy doing things spontaneously. | definitely  agree | slightly  agree | slightly  disagree | definitely  disagree |
| 35. | I am often the last to understand the point of a joke. | definitely  agree | slightly  agree | slightly  disagree | definitely  disagree |
| 36. | I find it easy to work out what someone is thinking or feeling just by looking at their face. | definitely  agree | slightly  agree | slightly  disagree | definitely  disagree |
| 37. | If there is an interruption, I can switch back to what I was doing very quickly. | definitely  agree | slightly  agree | slightly  disagree | definitely  disagree |
| 38. | I am good at social chitchat. | definitely  agree | slightly  agree | slightly  disagree | definitely  disagree |
| 39. | People often tell me that I keep going on and on about the same thing. | definitely  agree | slightly  agree | slightly  disagree | definitely  disagree |
| 40. | When I was young, I used to enjoy playing games involving pretending with other children. | definitely  agree | slightly  agree | slightly  disagree | definitely  disagree |
| 41. | I like to collect information about categories of things (e.g. types of car, types of bird, types of train, types of plant, etc.). | definitely  agree | slightly  agree | slightly  disagree | definitely  disagree |
| 42. | I find it difficult to imagine what it would be like to be someone else. | definitely  agree | slightly  agree | slightly  disagree | definitely  disagree |
| 43. | I like to plan any activities I participate in carefully. | definitely  agree | slightly  agree | slightly  disagree | definitely  disagree |
| 44. | I enjoy social occasions. | definitely  agree | slightly  agree | slightly  disagree | definitely  disagree |
| 45. | I find it difficult to work out people’s intentions. | definitely  agree | slightly  agree | slightly  disagree | definitely  disagree |
| 46. | New situations make me anxious. | definitely  agree | slightly  agree | slightly  disagree | definitely  disagree |
| 47. | I enjoy meeting new people. | definitely  agree | slightly  agree | slightly  disagree | definitely  disagree |
| 48. | I am a good diplomat. | definitely  agree | slightly  agree | slightly  disagree | definitely  disagree |
| 49. | I am not very good at remembering people’s date of birth. | definitely  agree | slightly  agree | slightly  disagree | definitely  disagree |
| 50. | I find it very easy to play games with children that involve pretending. | definitely  agree | slightly  agree | slightly  disagree | definitely  disagree |

**Developed by:**

**The Autism Research Centre**

**University of Cambridge**

© MRC-SBC/SJW Feb 1998
